# A new culture medium for recovering the agents of Cryptococcosis from environmental sources

**DOI:** 10.1590/S1517-838246220130726

**Published:** 2015-06-01

**Authors:** Dulcilena de Matos Castro e Silva, Dayane C.S. Santos, Sandra R.B.S. Pukinskas, Julia T.U. Oshida, Lidiane Oliveira, Anderson F. Carvallho, Márcia S.C. Melhem

**Affiliations:** Instituto Adolfo Lutz, Instituto Adolfo Lutz, Secretaria da Saúde, Governo do Estado de São Paulo, São Paulo, SP, Brasil, Instituto Adolfo Lutz, Secretaria da Saúde, Governo do Estado de São Paulo, São Paulo, SP, Brazil.

**Keywords:** *Cryptococcus*, isolation techniques, DRBC

## Abstract

The isolation of Cryptococcosis agents from environmental samples may be difficult due to the presence of groups of fast-growing fungi. We propose a new culture medium based on a modification of Dichloran Rose-Bengal Chloramphenicol Medium (DRBCm) to detect colonies of *Cryptococcus neoformans*. Our results indicate that DRBCm is superior to the classical Bird Seed Agar in its ability to detect colonies of *C. neoformans*.

Cryptococcosis, which is caused by *Cryptococcus* species, is considered an important systemic mycosis due to its severe pulmonary and central nervous system forms (Perfect and Casadevall, 2002). Most infections by *Cryptococcus* spp. are acquired by inhaling infectious propagules that are present in the environment (Lazera *et al.*, 2000). The association between clinical specimens and environmental samples was demonstrated by molecular studies (Delgado *et al.*, 2005). The environmental isolation of the two main species, *i.e.*, *C. neoformans* and *C. gattii*, in health surveillance studies, may reveal areas of permanent settlement or transitional areas that are at risk of exposing the population to these agents (Baltazar and Ribeiro, 2005; Granados and Castañeda, 2005; Kumar *et al.*, 2009; Refojo *et al.*, 2009).

Although not completely described, the known ecology of *Cryptococcus* spp. present in soil and vegetal materials allows us to speculate that these organisms may contribute to the decomposition of organic material (Fortes *et al.*, 2001; Yarwood *et al.*, 2010).

The presence of other microorganisms in high concentrations in the same environmental niche as *Cryptococcus* spp, such as filamentous fungi, has direct implications for obtaining pure cultures of the cryptococcosis agents (Henson, 1981; Lacaz *et al.*, 2002; Pedroso *et al.*, 2009; Soares *et al.*, 2005). The ability to simultaneously recover *Cryptococcus* spp. and other organisms depends on several factors, including the techniques, the fungal burden of each species and the culture media used (Alvarez *et al.*, 2003). Media containing substrates for the phenol oxidase enzyme, which is produced by *Cryptococcus* spp., have long been recommended for this activity. In the 1964, Staib proposed a culture medium containing Guizotia abyssinica seed extract, which allowed for the presumptive identification of this genre by its brownish pigmentation.

The isolation of some *Cryptococcus* spp. is quite difficult due to the presence of filamentous fungi, which are dispersed in all natural niches and grow quickly in media, thereby preventing the growth of yeast colonies. The rapid mold dispersion in the culture medium can be reduced using selective media. The Dichloran Rose-Bengal Chloramphenicol (DRBC) agar is a special medium for the isolation and enumeration of yeasts and molds and is commonly employed in analyses of food spoilage (King *et al.*, 1979). The compounds present in the medium limit fast-growing mold colonies, thus allowing for the concomitant growth of slow-growing yeasts, such as *Cryptococcus* spp. The ability of these genera to produce brown pigments on phenolic substrates could facilitate the visual distinction of colonies on agar medium among other yeast genera (Denning *et al.*, 1990; Eisenman *et al.*, 2009; Nosanchuk and Casadevall, 2003; Vidotto *et al.*, 2004).

Ideally, a medium that combines the restriction of airborne fungi colonies while enhancing the brown pigmentation of *Cryptococcus* would better enable the isolation of the agents of cryptococcosis from the environment.

This study proposes an improved medium for isolating *Cryptococcus* spp. from environmental vegetal samples. We propose a modified medium (DRBCm) prepared with an infusion of 50 g of Guizzotia absynnica seeds in 1000 mL of distilled water containing 2 g of pure creatinine compound and 15 g of DRBC commercially formulated agar. The performance of the DRBCm was compared with the classic *Guizzotia absynnica* (BSA) agar for recovering *C. neoformans* colonies. For this purpose, we performed two experiments. The first assay employed the artificial inoculation of *C. neoformans* cells into vegetal samples. Five standardized suspensions of *C. neoformans* ATCC 90112 strain-type were prepared and mixed with a single vegetal sample that was divided into four aliquots. Each vegetal aliquot was inoculated with a distinct fungal burden of 10^5^, 10^4^, 10^3^, 10^2^, or 10 cfu/mL. These inocula were prepared using an initial 10-mL saline suspension containing 0.5–2.5 × 10^6^ cfu/mL from a fresh 24-h culture of *C. neoformans* strain-type on Sabouraud dextrose agar.

The second assay used two positive hollow tree materials that were previously analyzed for the presence of *C. neoformans*. One of the positive samples, called the high cryptococcal burden sample, contained 2 × 10^4^ cfu/mL. The other positive sample, called the low cryptococcal burden, contained 1 × 10^2^ cfu/mL. Both the high and low cryptococcal burdens were processed in the same manner. Five grams of each positive sample was resuspended in a 20-mL solution, vortexed for 5 min at 150 rpm and centrifuged. Eight milliliters of the supernatant was mixed with a 2-mL solution of streptomycin-penicillin (4.5 mg/mL and 10 mg/mL, respectively). Each experiment was performed in triplicate. The data obtained from the experiments were evaluated using the statistically significant Fisher's exact test (Farmacopéia Brasileira, 2010; ANVISA, 2013). The resulting suspensions were kept for 20 min for bacterial decontamination. Next, a 10-μL loop was used to inoculate the DRBCm and bird seed agar medium, which were then distributed in 5 dish plates each. All experiments were performed in duplicate, and a negative control vegetal sample was used.

Colony counting was used to quantify the growth of melanized yeast colonies, non-melanized yeast colonies and mold colonies in each inoculated plate. A geometric mean of cfu/mL was obtained for the surface of all BSA and DRBCm medium dish plates. The presence of capsulated *C. neoformans* was confirmed in all melanized yeast colonies before the data were compiled*.*

Innumerable yeast species and filamentous fungi members are present in the same natural niches of *Cryptococcus* spp. (Steenbergen and Casadevall, 2000). The search for environmental isolates of *Cryptococcus* is not new, and recovering the cryptococcal colonies is a difficult task. Selective culture media that have high specificity, are easy to prepare, and have a reduced price are desired. To this end, many studies have been performed to formulate an effective agar for the primary presumptive identification of *Cryptococcus* members (Garcia-Rivera *et al.*, 2005; Gokulshankar *et al.*, 2011; Hernandez *et al.*, 2003; Menezes *et al.*, 2011; Mseddi *et al.*, 2011; Nandhakumar *et al.*, 2006; Pedroso *et al.*, 2009; Stepanovic *et al.*, 2002). Facing the challenger of recovering melanized colonies from environmental organic samples, we found intense fungal growth in both experiments, using either artificially inoculated vegetal debris or naturally infected hollow tree material. Both culture media yielded isolated colonies of *C. neoformans* and non-melanized yeast colonies in addition to mold colonies.

*C. neoformans* have a laccase, dipheniloxidase, which converts diphenolic compounds into melanin. Polacheck and Kwon-Chung (1988) were the first to detail the process of melanogenesis in *C. neoformans*. Some factors influence the synthesis of phenoloxidase, including the glucose and enzyme substrate concentrations, as previously demonstrated (Polacheck and Kwon-Chung, 1988). The use of natural products, such as bird seed, for infusion, as employed in this study, can lead to distinct results, depending on the enzyme substrate concentration, although testing different batches of agar confirmed the ability of the medium to pigment production of *Cryptococcus* spp (Nandhakumar *et al.*, 2006). *C. gattii* as C. *neoformans* has the same properties for the use of phenolic compounds and could showed pigmentation on bird seed media and DRBCm; however additional studies should be performed to evaluate the performance of both media in isolation *C. gattii* colonies.

In this sense, a chemically defined substrate for melanin production, such as L-DOPA and caffeic acid, is useful for controlling this issue. However, in this study, we were unable to obtain L-DOPA due to its high cost in comparison with the employed bird-seeds. The estimated price for the enzyme substrate needed to prepare one liter of agar is US$40.00 using L-DOPA, but this cost decreases to less than a dollar if bird-seed agar is employed (data not presented). This is indeed a limitation of the study, but this weakness is unlikely to affect the validity and applicability of the results in routine practice, particularly for many laboratories in developing countries that may face similar difficulties. The ability of *Cryptococcus* spp. to produce light to dark brown-colored colonies in the DRBCm agar containing creatinin and the bird-seed infusion presumptively identifies the genera. Nevertheless, we found at least 3 times as many colonies of *C. neoformans* in DRBCm as were found in the BSA medium (Table 1). Furthermore, the detection limit for DRBCm was 10^2^ cfu/mL, which is lower than the limit of 10^3^ cfu/mL observed for BSA medium. Fisher's exact test showed significant differences between DRBC agar and BSA agar (p < 0.05).

**Table 1:** Detection limit for recovering *C. neoformans* colonies using two media culture in experimental procedures.

CFU/mL plated	cfu/mL recovered	
	
	DRBCm agar	BSA agar
10^5^	1.5 × 10^3^	0.5 × 10^3^
10^4^	0.8 × 10^3^	0.1 × 10^3^
10^3^	0.4 × 10^3^	0
10^2^	0.1 × 10^3^	0
10^1^	0	0

Colonies of slower growing yeasts such as *Cryptococcus* could be prevented from growing due to competition for nutrients and space on the media, so we searched for a solution that would allow for the growth of such fungi. When we tested naturally contaminated material with a high cryptococcal burden, we found a higher geometric mean in DRBCm than in the BSA medium for *C. neoformans* (1.8 × 10^4^ cfu/mL and 1.3 × 10^4^ cfu/mL), non-melanized yeasts (0.25 × 10^4^ cfu/mL and 0.08 × 10^4^ cfu/mL), and molds (2.2 × 10^4^ cfu/mL and 0.92 × 10^4^ cfu/mL). For material infected with a low cryptococcal burden seeded in DRBCm and BSA medium, the results were more dramatic because no isolation of *C. neoformans* occurred in the BSA medium, which contrasted with the geometric mean of 0.6 × 10 cfu/mL that was verified for DRBCm (Figure 1). In fact, increasing the number of CFU analyzed increases the probability of isolating *C. neoformans*, according to previously reported data (Alvarez *et al.*, 2013). We did not obtain any growth of non-melanized yeasts in either medium, and we encountered similar results for molds, *i.e.*, 0.87 × 10^4^ cfu/mL and 0.91 × 10^4^ cfu/mL for the DRBCm and BSA media, respectively. We thought that DRBCm agar, due to its ability to limit the size of the colonies of fast-growing filamentous fungi, could contribute to minimizing this problem.

**Figure 1:**
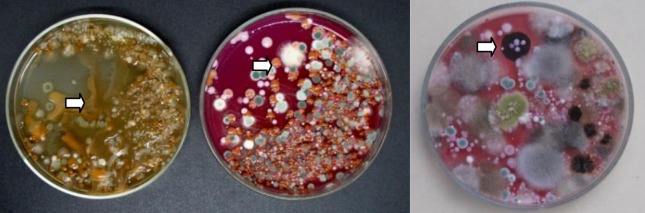
Recovering of *Cryptococcus neoformans* colonies (arrows) in: bird seed agar (left) and Dichloran Rose-Bengal Chloramphenicol modified agar (center) and Dichloran Rose-Bengal Chloramphenicol agar (right).

The DRBCm innovation tested in this study allowed colonies of melanized *Cryptococcus* cultures to be easily differentiated in environmental samples. Knowledge of formulating control strategies in natural niches. With monitoring data available, there may be a need for health authorities to implement hygiene measures, especially measures that reduce the environmental loads of yeast substrates that are favorable to their development.
